# Role of interventional radiology in the management of complications after pancreatic surgery: a pictorial review

**DOI:** 10.1007/s13244-014-0372-y

**Published:** 2014-12-17

**Authors:** Giovanni Mauri, Chiara Mattiuz, Luca Maria Sconfienza, Vittorio Pedicini, Dario Poretti, Fabio Melchiorre, Umberto Rossi, Fabio Romano Lutman, Marco Montorsi

**Affiliations:** 1 Servizio di Radiologia, IRCCS Policlinico San Donato, San Donato Milanese, Milano, Italy; 2 Dipartimento Aziendale di Oncologia, Struttura Complessa di Radiologia Oncologica Interventistica, Azienda Ospedaliera Ospedale di Circolo di Busto Arsizio, Varese, Italy; 3 Facoltà di Medicina e Chirurgia, Scuola di Specializzazione in Radiodiagnostica, Università degli Studi di Milano, Milano, Italy; 4 Dipartimento di Scienze Biomediche per la Salute, Università degli Studi di Milano, San Donato Milanese, Milano Italy; 5 Dipartimento di Diagnostica per Immagini, IRCCS Istituto Clinico Humanitas, Rozzano, Italy; 6 Dipartimento di Diagnostica per Immagini, Azienda Ospedaliera San Paolo, Milano, Italy; 7 Dipartimento di Scienze Diagnostiche, Azienda Ospedaliera San Carlo Borromeo, Milano, Italy; 8 Dipartimento di Chirurgia, Azienda Ospedaliera Humanitas (Humanitas Research Hospital), Rozzano, Italy; 9 Servizio di Radiologia, IRCCS Policlinico San Donato, Piazza Malan 2, 20097 San Donato Milanese, Milano Italy

**Keywords:** Interventional radiology, Duodenopancreasectomy, Complication, Embolisation, Biliary drainage

## Abstract

Pancreatic resections are surgical procedures associated with high incidence of complications, with relevant morbidity and mortality even at high volume centres. A multidisciplinary approach is essential in the management of these events and interventional radiology plays a crucial role in the treatment of patients developing post-surgical complications. This paper offers an overview on the interventional radiological procedures that can be performed to treat different type of complications after pancreatic resection. Procedures such as percutaneous drainage of fluid collections, percutaneous transhepatic biliary procedures, arterial embolisation, venous interventions and fistula embolisation are viable treatment options, with fewer complications compared with re-look surgery, shorter hospital stay and faster recovery. A selection of cases of complications following pancreatic surgery managed with interventional radiological procedure are presented and discussed.

*Teaching Points*

• *Interventional radiology is crucial to treat complications after pancreatic surgery*

• *Percutaneous drainage of collections can be performed under ultrasound or computed tomography guidance*

• *Percutaneous biliary procedures can be used to treat biliary complications*

• *Venous procedures can be performed effectively through transhepatic or transjugular access*

• *Fistulas can be treated effectively by percutaneous embolisation*

## Introduction

Pancreatic resection is a surgical procedure associated with significant morbidity and mortality even at specialised high-volume centres [1–3]. Although in recent years refinements in surgical technique and perioperative management have led to a reduction in perioperative mortality, the incidence of postoperative complications (including intra-abdominal abscesses and leakages [4–9], biliary complications [10–14] and vascular complications [15–18]) still remains high. Moreover, in case of complications, surgical re-operation is associated with a high mortality rate [1–3]. In a large series of 650 patients, Yeo et al. [2] reported an overall mortality of 1.4 % and a morbidity of 41 %, with a mean length of hospital stay of 13 days. They reported re-operation in 26/650 patients (4 %) and identified the absence of reoperation as an independent predictor of prolonged survival [2].

Interventional radiology (IR) provides a minimally invasive alternative for managing post-surgical complications [19, 20]. Several different IR procedures, such as percutaneous drainage, aspiration of abscesses or fluid collections [4, 5], percutaneous transhepatic biliary drainage [6], and arterial embolisation [21–23] have been introduced in clinical practice to treat post-surgical complications. IR procedures are an alternative approach to manage post-surgical complications less invasive than surgical re-intervention, and may lead to a reduction in hospital stay and re-operation rate [10, 11].

In the present pictorial review, we offer an overview on the IR procedures that can be performed to treat different types of complications after pancreatic resection.

## Percutaneous drainage

Intra-abdominal collections and abscesses represent the most common complication following pancreatic surgery [1–3]. Once an intra-abdominal collection is identified, in most cases it is possible to place a percutaneous drainage under image guidance. When the collection is well visible using ultrasound (US), US-guided percutaneous drainage placement is generally the preferred choice, as US is widely available, easy to handle and allows for real-time monitoring of the drainage placement, being also free from ionising radiation [24]. When the collection is located deep in the abdomen and is not well seen at US, computed tomography (CT) generally offers good anatomical definition to guide the safe placement of a percutaneous drainage. Drainage placement can be performed using the trocar or Seldinger technique. In the trocar technique, the drainage catheter containing a trocar needle is inserted directly into the collection. The trocar technique provides a fast deployment of the drainage that can be extremely helpful in critically ill or agitated patients. The Seldinger technique implies multiple steps: the collection is punctured with a small calibre needle, different calibre guidewires are inserted and the drainage is then advanced up to the collection over the guidewire. The Seldinger technique is particularly helpful when there is a small window to reach the collection, as typically happens when a retroperitoneal collection has to be drained through an anterior approach. The main disadvantage of this technique is that it may require considerably longer time than the trocar technique. The correct location of the needle or drainage can be confirmed by aspiration of a small amount of material. Furthermore, a small amount of iodinated contrast may be injected through the catheter to identify the presence of an underlying fistula.

In a series of 373 subjects who underwent pancreaticoduodenectomy reported by Zink et al. [5], percutaneous drainage was required in 22.2 % of patients after surgery. They report an immediate technical and overall success rate of 97.6 and 79.6 %, respectively.

**Fig. 1:**
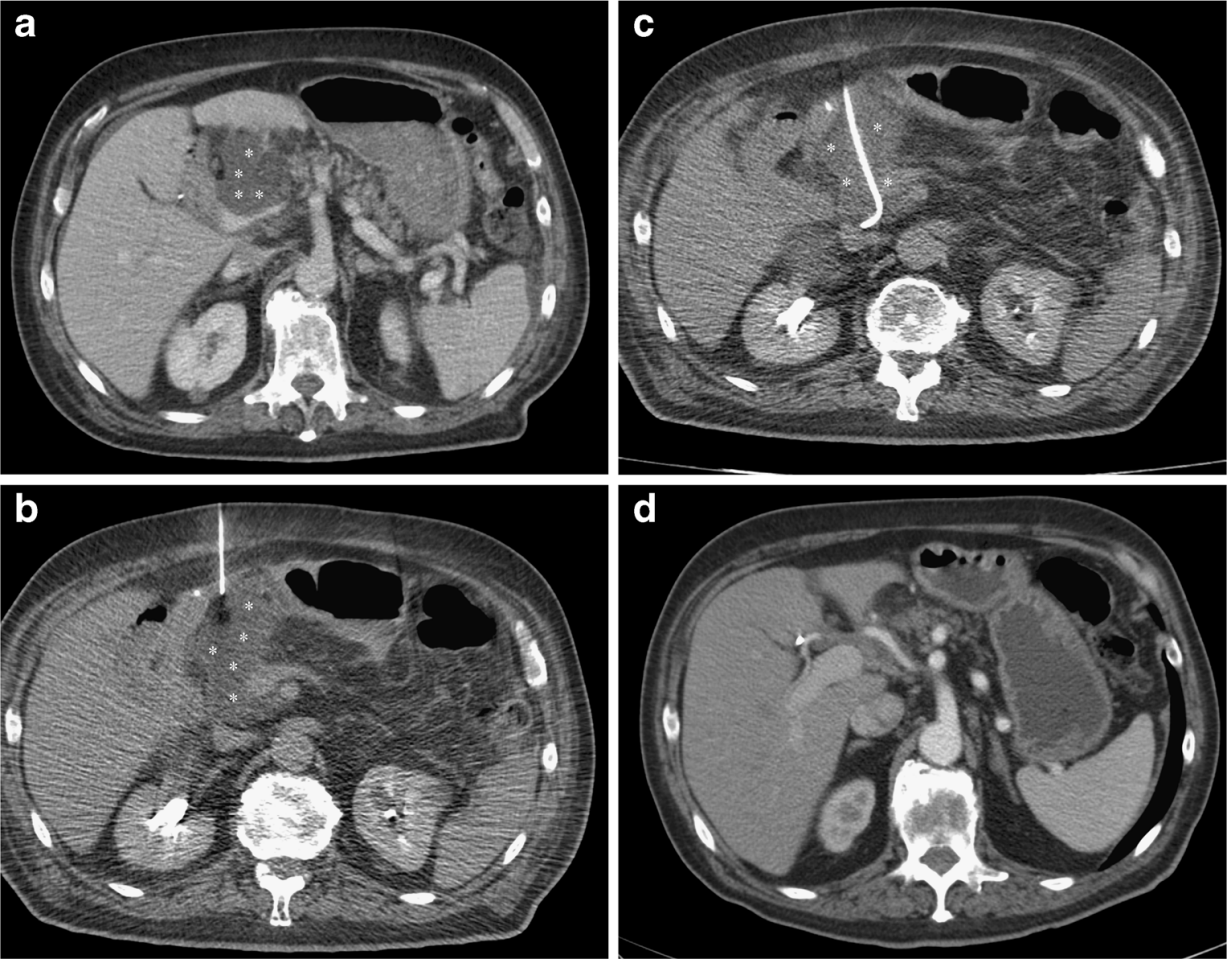
Use of a percutaneous drainage to treat a post-pancreaticoduodenectomy fluid collection. **a** CT scan shows post-pancreaticoduodenectomy retroperitoneal fluid collection (*asterisks*). **b** The fluid collection (*asterisks*) is punctured with a small needle under CT guidance. **c** A percutaneous drainage (*arrow*) is inserted into the collection (*asterisks*) using the Seldinger technique. **d** At the end of the treatment complete resolution of the collection is obtained

A case of a patient with fluid collection after pancreaticoduodenectomy successfully managed with percutaneous CT guided drainage is shown in Fig. 1.

## Percutaneous transhepatic biliary procedures

In patients treated by pancreaticoduodenectomy with Roux-en-Y bilio-enteric reconstruction, endoscopical access to the biliary tree is not feasible. Thus, in this kind of patient, a percutaneous transhepatic route is the only approach to the biliary system in case of biliary complications. The access to the biliary system is obtained by puncturing a peripheral bile duct under US and/or fluoroscopic guidance.

**Fig. 2:**
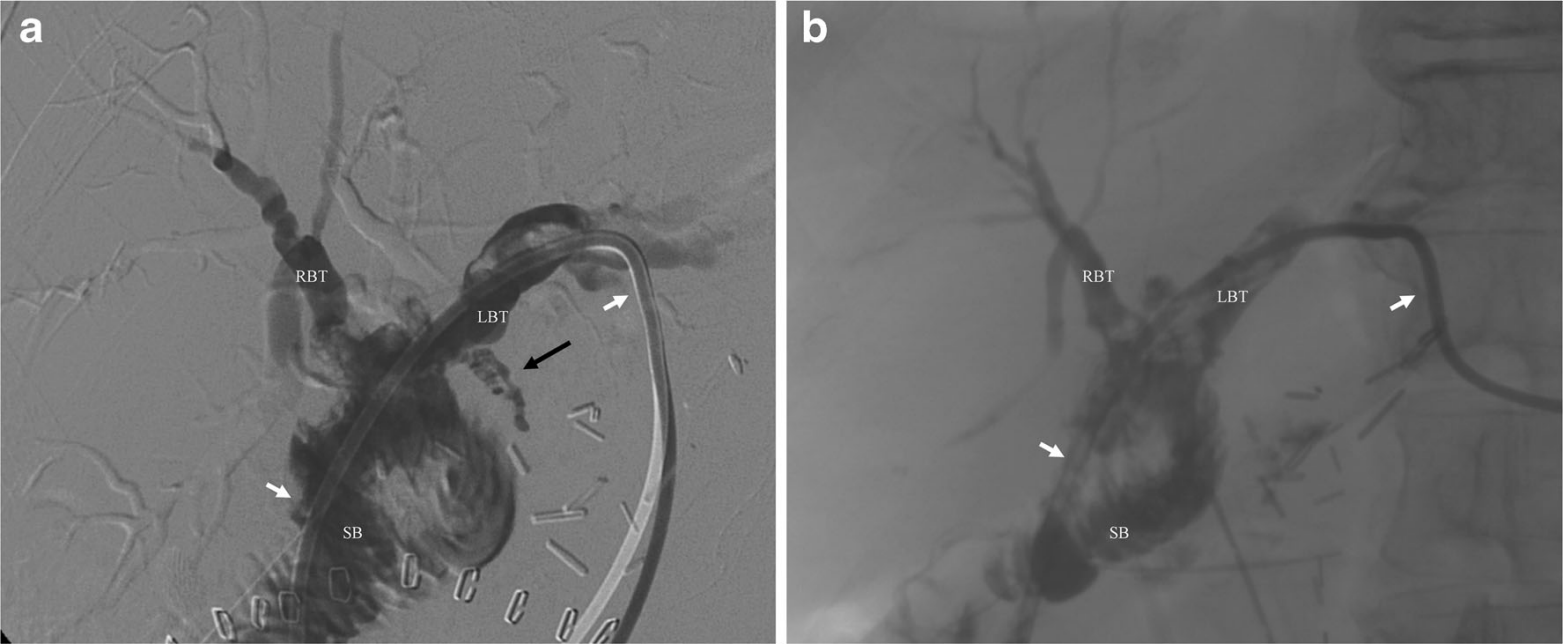
Use of a percutaneous transhepatic biliary drainage to treat a post-surgical biliary leak. **a** Percutaneous colangiography demonstrates a biliary leak (*arrow*) and a biliary drainage (*white arrows*) is inserted via segment III left lobe approach; *RBT* right biliary tree, *LBT* left biliary tree, *SB* small bowel. **b** Final result with complete healing of the leak and absence of contrast leakage; *RBT* right biliary tree, *LBT* left biliary tree, *SB* bowel

Percutaneous biliary procedures can be particularly helpful in postoperative biliary leaks. They have been reported in about 3–4 % of cases after pancreatic surgery [1–3]. These complications are often associated with others, in particular with pancreatic fistula and fluid collections. In patients who develop such complication after surgery, percutaneous transhepatic biliary drainage—by allowing for a bile diversion from the site of the fistula—has been proven to be feasible and effective in the majority of cases [6, 20, 21]. The positioning of an occlusion balloon to obstruct the biliary duct above the site of the fistula, by allowing for complete external drainage of the bile, may represent a valuable option to treat post-surgical biliary leaks [7, 10]. A different and more recent technique is to use a covered stent that can close the bile leak and subsequently be retrieved percutaneously. Gwon et al. [25] used this approach successfully in 11 patients with postoperative bile leak, with no recurrence in the 1-year follow-up. A case of a patient with post-surgical biliary leak successfully treated with percutaneous transhepatic biliary drainage is shown in Fig. 2.

After bilio-pancreatic surgery, stricture of a biliary duct may represent a serious complication, [26]. Surgical re-intervention is associated with morbidity and mortality rates as high as 28 and 2.6 % respectively [26]. Percutaneous treatments represent an effective alternative to surgery in the treatment of such complication [27–29]. To achieve resolution of the stenosis, subsequent larger biliary drainage catheter may be inserted and left in place and balloon dilation can be performed. However, recurrence of stenosis may occur in up to 29–58 % of cases [27–29], and multiple treatment sessions may be required. Stents are rarely used in the treatment of benign strictures, as they have to be removed after a period because the tube itself may stimulate inflammatory reaction, fibrosis and stone formation. A novel option may be represented by the use of biodegradable biliary stents, which may improve the long-term patency rate, without requiring a subsequent procedure for removal [13].

**Fig. 3:**
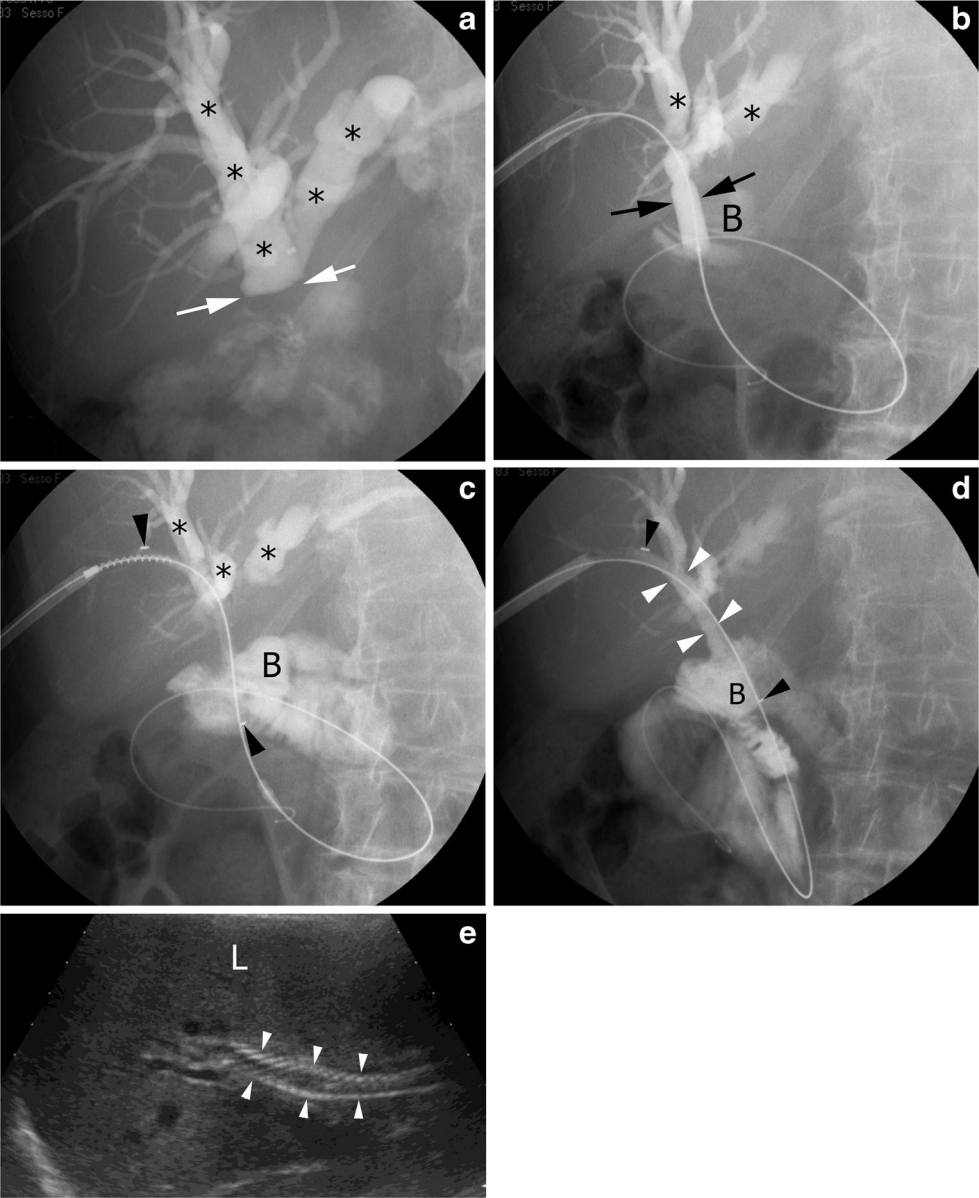
Percutaneous treatment of a benign stricture of the common bile duct by a bioabsorbable biliary stent. **a** Percutaneous transhepatic cholangiography demonstrates remarkably dilated bile ducts (*asterisks*). The obstruction can be clearly spotted (*white arrows*). **b** Bilioplasty procedure. The balloon (*black arrows*) is fully inflated to dilate the stricture. Radiopaque contrast agent can be detected in the bowel (*B*); *asterisks* dilated bile ducts. **c** A bioabsorbable stent is positioned to keep the stricture open. The stent is radiolucent, but two platinum, radiopaque markers can be seen (*black arrowheads*); *asterisks* dilated bile ducts, *B* bowel. **d** The stent is fully expanded and radiopaque bile can be seen flowing through the patent common bile duct (*white arrowheads*); *black arrows* radiopaque stent markers; *B* bowel. **e** US follow-up at 3 months demonstrating good visibility of the stent (*arrowheads*) that was correctly expanded, and no dilation of the intrahepatic biliary ducts; *L* liver

A case of a patient with a post-surgical biliary stricture successfully treated with balloon dilation and placement of a biodegradable biliary stent is shown in Fig. 3.

## Arterial embolisation

Postoperative intra-abdominal arterial haemorrhage is still one of the most serious complications, with a reported incidence between 1.5 and 15 %, and a reported mortality rate of 20–50 % [15–17]. Haemorrhage is defined as ‘early’ when occurring within 24 h after surgery, while ‘late’ haemorrhages occur after 24 h [30]. Early haemorrhage requires immediate laparotomy, as it is generally caused by a technical failure or underlying coagulopathy, while the most appropriate management of delayed haemorrhage still remains controversial. In haemodynamically stable patients, CT is crucial to address the suspicion of a delayed haemorrhage and is extremely useful prior to angiography as it may avoid it or guide it. In unstable patients, direct visceral angiography has been suggested as the best method to elucidate the site of bleeding, with the advantage of sparing time for a subsequent immediate intra-arterial treatment.

Transcatheter arterial embolisation has been reported to be safe and effective, with a reported success rate of 50–100 %, gaining acceptance for the treatment of intra-abdominal bleeding [15–17]. Once the site of bleeding has been identified, several different techniques may be used to stop bleeding and achieve haemostasis. In case of terminal vascularisation, a proximal embolisation of the bleeding vessel may be enough to achieve the haemostasis, while in the presence of collaterals, it is crucial to embolise both the inflow and outflow vessels in order to avoid re-bleeding (“isolation technique”) [22]. Different materials are nowadays available to obtain vascular occlusion, and have to be chosen according to the desired type of vascular occlusion (transient or permanent). Transient embolisation, generally required due to a traumatic haemorrhage, is reached by resorbable materials, which allows for restoring the blood flow in a variable window of time (inter alia: autologous blood clot, gelatine or fibrin sponge). The main advantage of such materials is to avoid definitive occlusion of the treated vessel. However, a non-negligible risk of re-bleeding has to be considered once the material has been absorbed. Permanent embolisation is reached using non-resorbable materials (polyvynilic alcohol, bucrilate, metallic coils or detachable balloons) that induce a permanent vessel occlusion. At the present, an ideal material does not exist, thus the choice of the most appropriate embolic material and technique for embolisation, crucial to minimise failure and complications, requires the presence of a IR team with high experience and the availability of several different materials.

Stent grafting of the artery at the site of bleeding has been proposed as an alternative or in addition to embolisation, with the advantage of maintaining the patency of the end organ, thus minimising the risk of ischaemia deriving from embolisation [21]. A recent meta-analyisis comparing laparotomy and transarterial embolisation for the management of delayed postoperative haemorrhage found a reduction in mortality (43 % vs 20 %) and morbidity (77 % vs 35 %) in the IR group, even if not reaching the statistical significance. Authors conclude that the appropriate treatment pathway for late haemorrhage ultimately will be decided by the clinical status of the patient and the institution preference [16].

**Fig. 4:**
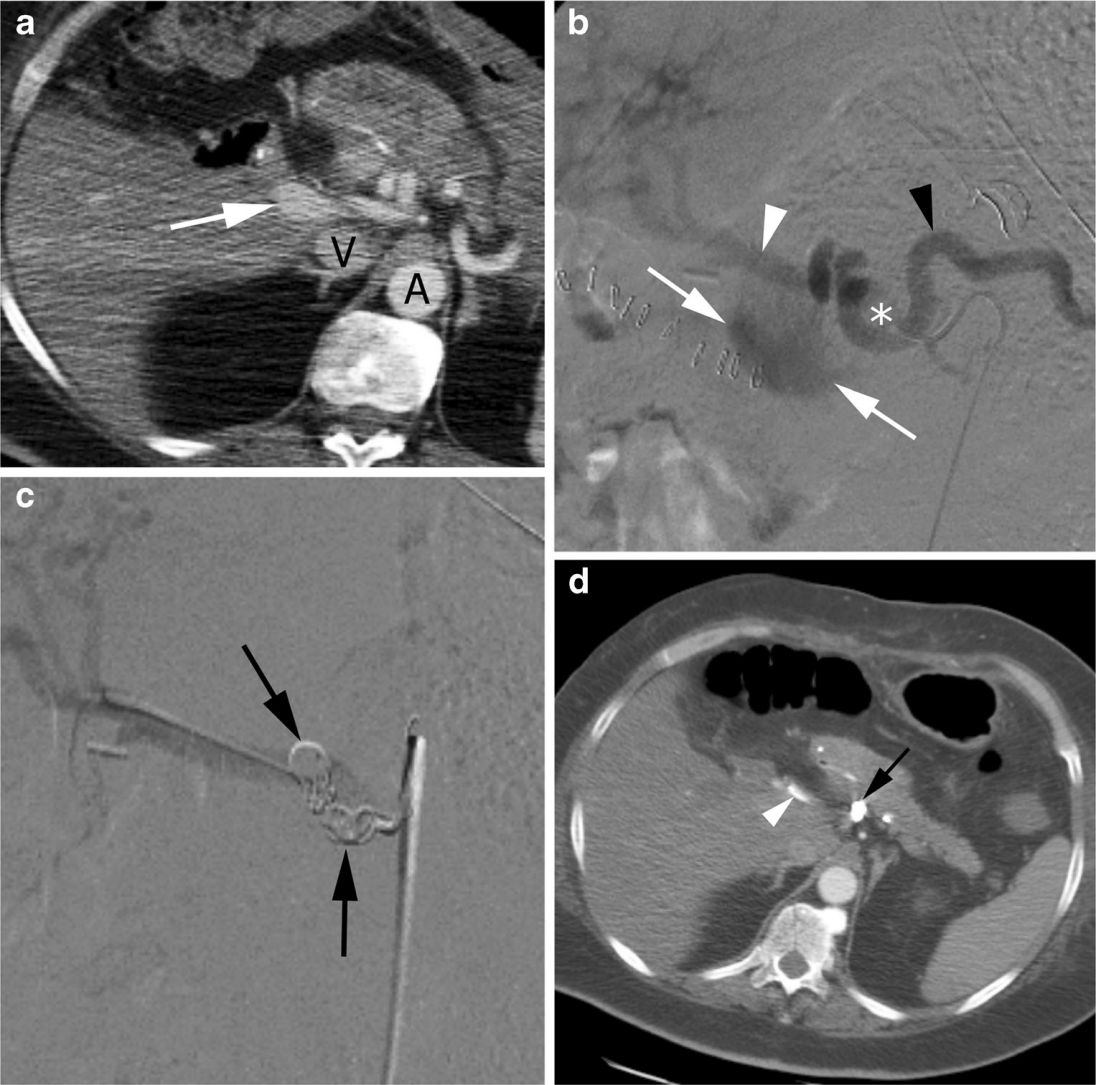
Arterial embolisation of a post-surgical haemorrhage. **a** CT scan showing contrast extravasation (*arrow*) from the celiac trunk at the level of the bifurcation between hepatic and splenic artery; *V* inferior vena cava, *A* abdominal aorta. **b** Angiographic appearance of the active bleeding with contrast extravasation (*arrow*s); *asterisk* celiac trunk, *black arrowhead* splenic artery, *white arrowhead* hepatic artery. **c** Angiography after embolisation with coils (*arrows*) demonstrating absence of contrast extravasation at the level of previous bleeding. **d** CT scan at 2 years, demonstrating absence of contrast extravasation at the level of previous bleeding and preserved patency of proper hepatic artery (*white arrow*), even in the presence of part of a coil in its lumen (*arrowhead*); *black arrow* clustered metallic coils in the focus of arterial lesion properly embolised

A case of transcatheter arterial embolisation for a post-surgical haemorrhage is shown in Fig. 4.

## Venous interventions

In some cases, post-surgical complications may involve the portal and mesenteric veins, which may develop stenosis or thrombosis after surgical treatment. Moreover, with the recent advancement of vascular reconstruction with polytetrafluoroethylene graft, this occurrence may happen more frequently in the future. Percutaneous endovascular treatment, such as transjugular portosystemic shunt, direct and indirect thrombolysis, stenting and mechanical thrombectomy, described mainly in liver-transplant patients, may represent a valuable option in the management of these conditions after pancreatic surgery [2, 5, 18]. The access to the portal system may be achieved through a transhepatic route, with the direct image guided puncture of a peripheral portal branch, or through a transjugular approach, by puncturing the portal system from the hepatic veins. Transhepatic access is generally easier than transjugular access and represents the first choice strategy in patients with normal coagulation parameters. In patients with thrombosis of the portal or mesenteric vein, in which anticoagulation treatment has been undertaken, the best choice is represented by the transjugular approach, in order to minimise the risk of peritoneal haemorrhage correlated with direct liver puncture [18]. Once the access to the portal system has been achieved, it is possible to perform balloon dilation and stenting of post-surgical strictures using the several different commercially available devices. In case of thrombosis, direct trombo-aspiration through a catheter or infusion of trombolitic agents has been reported as a feasible and effective treatment [18].

**Fig. 5:**
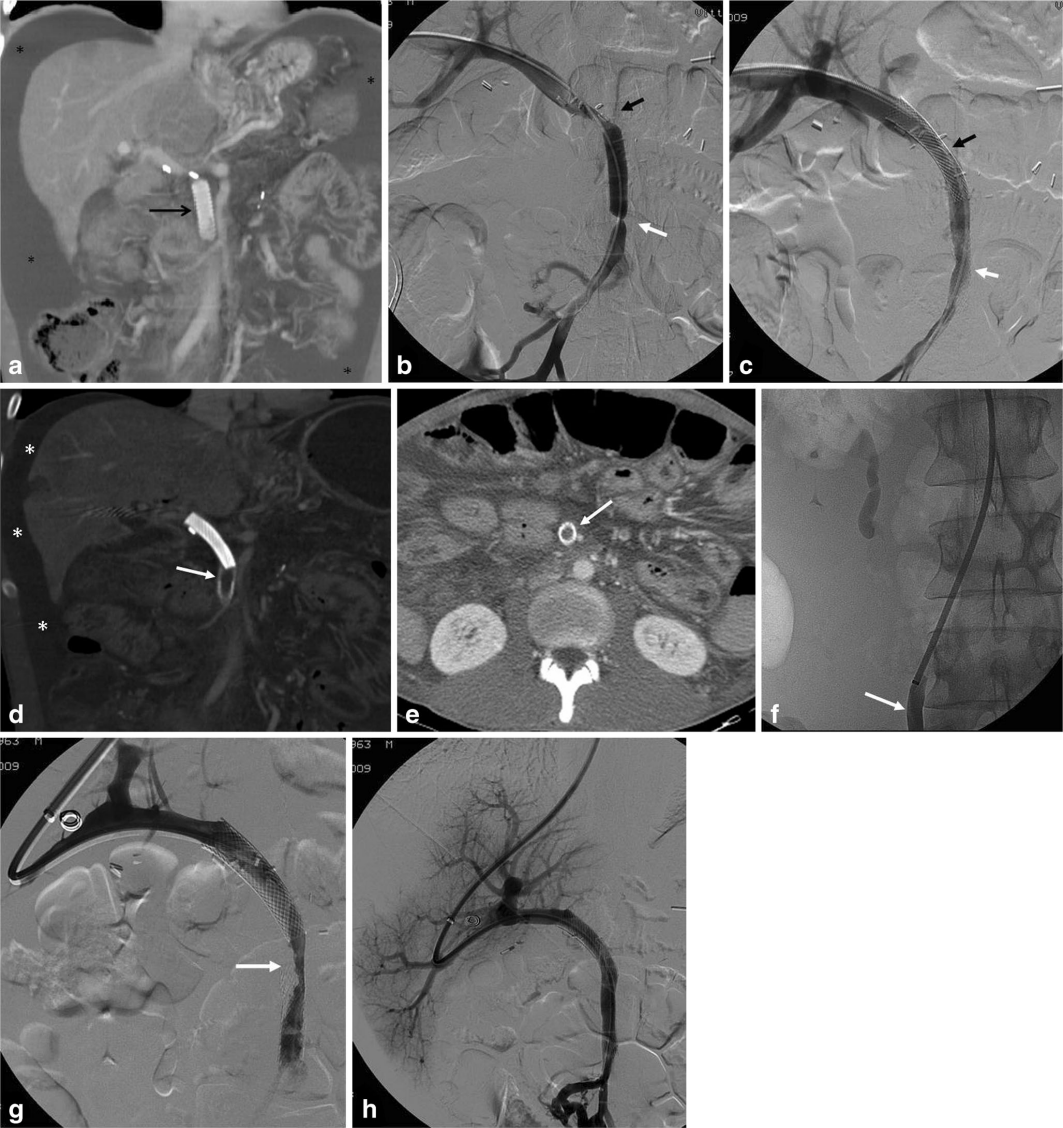
Treatment of a patient with postoperative mesenteric vein stenosis and thrombosis by percutaneous transhepatic stenting, thromboaspiration, and thrombolysis through a transjugular approach (the case has been partially previously reported [18]). **a** Maximum intensity projection reconstruction of abdominal MDCT shows surgical vascular graft (*black arrow*) at the level of the superior mesenteric vein and ascitic fluid (*asterisks*) **b** Percutaneous transhepatic angiography demonstrates stenosis at the level of proximal (*black arrow*) and distal (*white arrow*) anastomoses of the PTFE graft. **c** Angiography after stent placement (*black arrow* proximal stent, *white arrow* distal stent) demonstrating resolution of the stenosis. **d** MDCT MIP reconstruction and **e** axial image showing intra-stent thrombosis (*white arrow*) and ascitic fluid (*asterisks*). **f** Contrast injection in SMV (*arrow*) demonstrates complete vessel occlusion. **g** Angiography after aspiration thrombectomy and angioplasty shows residual thrombus (*arrow*). **h** Complete resolution and calibre restoration after direct thrombolysis with rt-PA

A case of post-surgical mesenteric vein stenosis and thrombosis treated percutaneously is shown in Fig. 5.

## Fistula embolisation

Fistulas are the most common complication following pancreatic surgery after presence of fluid collections [1–3]. The most frequent type is pancreatic fistula, followed by enteric and biliary fistulas [1–3]. This kind of complication is frequently associated with others, in particular the presence of an intra-abdominal fluid collection. Recent reports highlighted how this kind of complication can be successfully managed without surgery in over 90 % of patients [9]. In the non-operative management of such complications, IR plays a crucial role.

In presence of a fluid collection, a percutaneously placed drainage may be enough to treat the collection, and in several cases to allow for the spontaneous closure of the fistula [4, 5]. If the main component of a fistula is biliary material, the transhepatic insertion of a biliary drainage, by diverting the bile from the site of the fistula, may be enough to determine the fistula closure. In cases with persisting biliary leak, the placement of an occlusion balloon above the fistula, by interrupting completely the bile flow towards the site of the fistula, has been reported as an effective tool [7–10].

Some authors reported the direct embolisation of the site of the fistula as a feasible and effective procedure. Reaching perfectly the site of the fistula is crucial to perform a correct procedure. This can be done through a previously placed surgical drainage, through an image-guided percutaneously placed drainage, or even through a transhepatic approach. Once the site of the fistula has been reached, several different materials can be used to perform embolisation, including ethanol, particles or different kind of glues [31–33]. Inter alia, cyanoacrilic glues seem to represent optimal materials for fistula percutaneous treatment, due to their high adhesive and haemostatic properties and fast polymerisation [31].

**Fig. 6:**
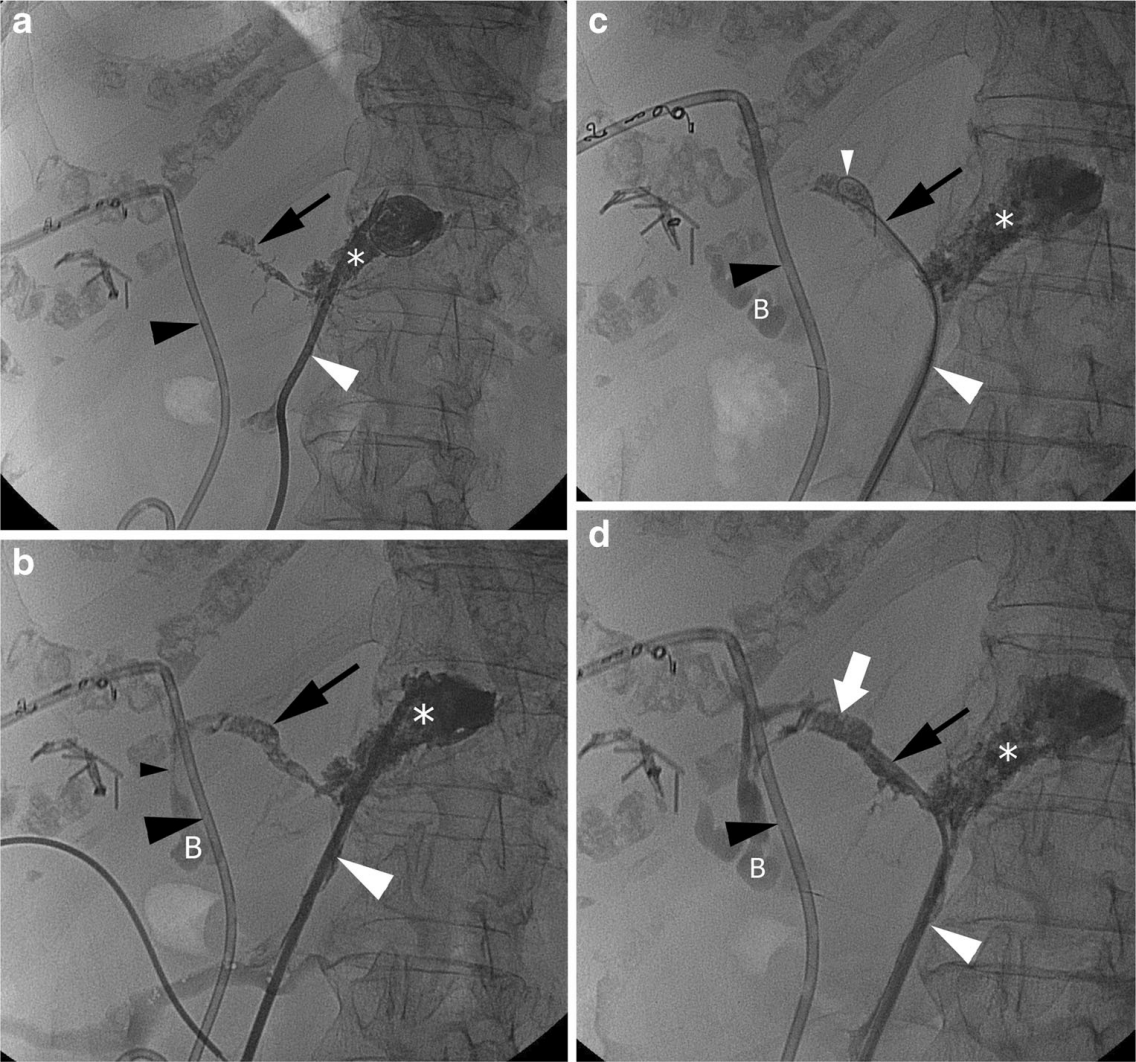
Successful percutaneous embolisation with cyanoacrilic glue of a postoperative fistula. **a**, **b** Contrast injection through a previously placed percutaneous drainage (*white arrowhead*) at the level of a fluid collection (*asterisk*) demonstrating a fistula (*black arrow*) with a communication (*small black arrowhead*) with the biliary system and bowel (*B*); *large black arrowhead* percutaneous transhepatic biliary drainage. **c** A guidewire (*small white arrowhead*) is advanced through the percutaneous drainage (*large white arrowhead*) into the fistula (*black arrow*); *asterisk* fluid collection, *black arrowhead* percutaneous transhepatic biliary drainage, *B* bowel. **d** A microcatheter (*white arrowhead*) is advanced over the wire at the level of the fistula (*black arrow*), and glue is injected (*white arrow*); *asterisk* fluid collection, *black arrowhead* percutaneous transhepatic biliary drainage, *B* bowel

A case of embolisation of a fistula with cyanoacrilic glue is shown in Fig. 6.

## Conclusions

IR plays an increasing, crucial role in the multidisciplinary management of complications after pancreatic surgery, providing a minimally invasive therapy also in critical patients, reducing recovery times and avoiding re-operation morbidity. IR procedures such as percutaneous drainage of fluid collections, percutaneous transhepatic biliary procedures, arterial embolisation, venous interventions and fistula embolisation are viable treatment options and have been reported as feasible, safe and effective techniques with fewer complications compared with re-look surgery, with a shorter hospital stay and faster recovery in the management of complications after pancreatic surgery.
